# From E-Waste to Hydrogen Evolution: Harnessing Recycled Precious Metals for Catalytic Efficiency in Hydrogen Evolution Reactions

**DOI:** 10.3390/ma16206795

**Published:** 2023-10-21

**Authors:** Stefan Mitrović, Snežana Brković, Sanja Živković, Nikola Zdolšek, Mina Seović, Jelena Georgijević, Ivana Perović

**Affiliations:** Department of Physical Chemistry, “Vinča” Institute of Nuclear Sciences—National Institute of the Republic of Serbia, University of Belgrade, Mike Petrovića Alasa 12–14, 11351 Belgrade, Serbia; stefan.mitrovic@vin.bg.ac.rs (S.M.); snezana.miulovic@vin.bg.ac.rs (S.B.); sanjaz@vin.bg.ac.rs (S.Ž.); zdolsek@vin.bg.ac.rs (N.Z.); mina050@vin.bg.ac.rs (M.S.); georgijevic@vin.bg.ac.rs (J.G.)

**Keywords:** e-waste, hydrogen evolution reaction, electrodeposition, CPU recycling, catalytic efficiency

## Abstract

Against the background of escalating global electronic waste (e-waste) and its rich reservoir of elements, this research addresses the exploitation of precious metals from discarded CPUs for potential applications in hydrogen production. The study systematically explores the influence of varied CPU sample preparation techniques on the formation of an electrode’s catalytic layer and the kinetics of the hydrogen evolution reaction (HER) in alkaline media. Four distinct e-waste samples, each subjected to different preparation protocols, were employed as sources in electrodeposition baths. The electrocatalytic efficiency of the resulting electrodeposited cathodes was evaluated, with the AR-CPU-1.4M electrode demonstrating superior properties. Morphological insights from SEM, coupled with elemental data from EDS and ICP analyses, revealed the intricate relationship between sample preparation, electrode characteristics, and HER kinetics. Notably, gold deposits and a prominent copper concentration emerged as defining attributes of our findings. This research underscores the potential of e-waste-derived metals, particularly in hydrogen production, providing an avenue for sustainable metal recovery and utilization.

## 1. Introduction

In today’s technological era, marked by rapid innovation and growing consumer demands, the electronics sector is on a growth trajectory rarely seen in other industries. However, this growth has been accompanied by a large-scale challenge: the escalation of electronic waste, or e-waste [1]. Data collated in 2019 highlight this alarming trend, pinpointing the global accumulation of electronic and electrical waste at an astounding 53.6 million metric tons—a surge of about 17.16% from the 44.4 million metric tons observed in 2014 [2]. With projections estimating this to touch 65.3 million metric tons by 2025, of which 11.4 million metric tons can be attributed to IT and telecommunications equipment and computers, the magnitude of the issue becomes evident [2,3]. Delving into the composition of these discarded devices reveals an abundance of elements, including an impressive 70 elements from the periodic table. Precious metals such as gold, silver, and copper dominate the mix, but a litany of other metals, including aluminum, nickel, and titanium, also make their presence felt [4]. Given that the Earth’s metal reserves are definitely limited and conventional mining is notoriously harmful to the environment [5], the argument for extracting these metals from e-waste becomes compelling. Such efforts not only support sustainability but could also act as the genesis of a transformative sector within the circular economy [6].

Our work postulates one possible application: the utilization of recycled metals, especially precious ones from e-waste, in the field of hydrogen production. Given the well-documented catalytic efficiency of noble metals in the hydrogen evolution reaction and other reactions of interest in energy converting applications [7,8], this exploration gains significant traction. A pivotal section of this paper will be devoted to understanding how the preparation nuances of recycled e-waste samples influence both the catalyst layer and its overarching activity.

The main goal of this research is to examine in detail the influence of variations in the preparation of samples from recycled CPUs (central processing units) on the formation of the catalyst layer and the kinetics of the hydrogen evolution reaction (HER) in alkaline media. In this effort, we applied four different e-waste samples, each subjected to different preparation protocols, to serve as sources in electrodeposition baths. The electrocatalytic efficiency of the resulting electrodeposited cathodes was investigated in detail through an in-depth analysis of the underlying mechanisms governing HER. Furthermore, a comprehensive examination of the morphology and compositional attributes of the cathodes was conducted to delineate the correlation between the preparation methodologies and the structural properties of the electrodeposited layers. Simultaneously, dynamic changes within the composition of the electrodeposition bath throughout the electrodeposition sequence were monitored to observe their consequent effects on the overall deposition process and subsequent HER kinetics in alkaline solutions. Through this multifaceted analysis, this study aims to illuminate the interdependencies between e-waste-derived sample preparation, the characteristics of the electrodeposited layers, and their collective consequences on the hydrogen evolution process.

## 2. Materials and Methods

### 2.1. Sample Collection, Pre-Treatment, and Preparation

All computer processors, CPUs, employed in this study were of the same brand and generation. The computers were disassembled, and the processors were carefully extracted from sockets and motherboards. Following extraction, the processors underwent cleaning to remove dirt, plastic, and thermal paste. This step was consistent across all sample types.

For the sample preparation protocols, four different sample preparation methods were used with the following designations:

- BM-CPU-1.4M: CPUs were mechanically fragmented and placed in a handmade laboratory tungsten carbide ball mill for a grinding duration of 6 h. Due to the CPUs’ inherent hardness, only a fraction of fine powder was derived. This powder’s particle sizes were measured using a Retsch AS200 Control lab sieve [9]. A total of 0.75 g of this powder was leached in a 10 mL solution consisting of 0.6 g sodium-chloride, 9.5 mL 1.4 M hydrochloric acid, and 0.5 mL 30% hydrogen peroxide for 1 h at room temperature.
- EM-CPU-1.4M: CPUs, removed from their metal cooling cases and with silicon boards segmented, were processed using an IKA A10 basic electrical mill (IKA-Werke GmbH & Co.KG, Staufen, Germany). The resultant powder displayed some heterogeneity in particle size, which was subsequently gauged using the Retsch AS200 Control lab sieve (Retsch GmbH, Haan, Germany). This powder was then subjected to the aforementioned leaching process.
- HM-CPU-1.4M: A CPU, once detached from its metal case, had its PCB (printed circuit board) partitioned into smaller sections to accommodate an MRC DMP-100 disc mill (MRC Laboratory-Instruments, Holon, Israel). After milling, particle size was evaluated, and the powder was processed using the specified leaching solution like in previous samples.
- AR-CPU-1.4M: This method involved a chemical preparation technique utilizing aqua regia (3:1 volume ratio of hydrochloric acid to nitric acid) (Merck KGaA, Darmstadt, Germany). In total, 60 mL of this solvent was employed to treat the CPU for 98 h. Post dissolution, undissolved residues were separated, and the remaining solution was evaporated down to a paste, washed thrice with hydrochloric acid, and finally reconstituted with deionized water. This yielded a metal-rich aqueous solution which was then adapted for electrochemical sedimentation bath preparation.

### 2.2. Electrodeposition and Electrochemical Testing

Metal electrodeposition baths, denoted the same as the prepared samples described above, were an aqueous mixture of 0.6 g sodium-chloride, 9.5 mL 1.4 M hydrochloric acid, and 0.5 mL 30% hydrogen peroxide (all analytical-grade chemicals from Merck KGaA, Darmstadt, Germany) with 0.75 g of powdered samples. Electrochemical testing solution was a 6 M KOH (p.a., Merck KGaA, Darmstadt, Germany) constituted using deionized water.

Electrodeposition was conducted in a standard two-electrode glass cell. The working electrode employed was high-purity (99.9%) metallic nickel with a surface area of 1 cm^2^, while the counter electrode was a high-surface-area platinum mesh. The electrodeposition process was carried out using Gamry 1000E Potentiostat (Gamry Instruments, Warminster, PA, USA) in the galvanostatic mode at current density of 10 mAcm^−2^ for two hours with constant stirring maintained at 300 rpm using a magnetic stirrer IKA C-MAG HS10 (IKA-Werke GmbH & Co.KG, Staufen, Germany).

Electrochemical evaluations were performed in a three-electrode glass cell. The working electrode comprised the metallic nickel with electrodeposited catalysts denoted the same as the samples from which they were obtained. The counter electrode remained a high-surface-area platinum mesh, and the reference electrode was an SCE (saturated calomel electrode). Prior to testing, the electrolyte was purged with hydrogen for 15 min. Electrochemical characterization consisted of recording and analyzing the polarization curves and EIS (Electrochemical Impedance Spectroscopy) spectra in order to determine HER (hydrogen evolution reaction) kinetics on electrodeposited catalysts. All electrochemical measurements were performed using a Gamry 1000 E Potentiostat (Gamry Instruments, Warminster, PA, USA). Polarization curves were recorded by changing the electrode potential in the range from −1.6 V to −0.6 V, with respect to the SCE reference electrode, at a rate of 1 mVs^−1^. Impedance spectra were recorded in the frequency range from 0.01 Hz to 100 kHz using an alternating signal with an amplitude of 10 mV superimposed on a constant overvoltage value in a range from −50 mV to −200 mV. Before each measurement, the working electrode was conditioned for 20 min at a constant current of 100 mA. The temperature of the solution in the electrochemical cell was 298 K.

The morphology and elemental analysis of the best-performing catalyst was investigated through scanning electron microscopy (SEM) analysis. The SEM micrographs were obtained using SEM FEI Scios2 Dual Beam System instrument (Thermo Fisher Scientific, Waltham, MA, USA) under high vacuum conditions and accelerating voltage of 10 kV with the applied magnification being up to 50,000×. The corresponding EDS (Energy-Dispersive X-ray Spectroscopy) spectra were captured utilizing the same instruments. Elemental composition analysis of electrodeposition baths before, during, and after deposition process were conducted via ICP-OES (Inductively Coupled Plasma Optical Emission spectroscopy) analysis using a Thermo Scientific ICP-OES 7000 series instrument (Thermo Fisher Scientific, Waltham, MA, USA).

All methods and protocols were carefully followed, ensuring consistency and reproducibility of results across all samples.

## 3. Results

### 3.1. Sample Preparation and Characterization

**Figure 1:**
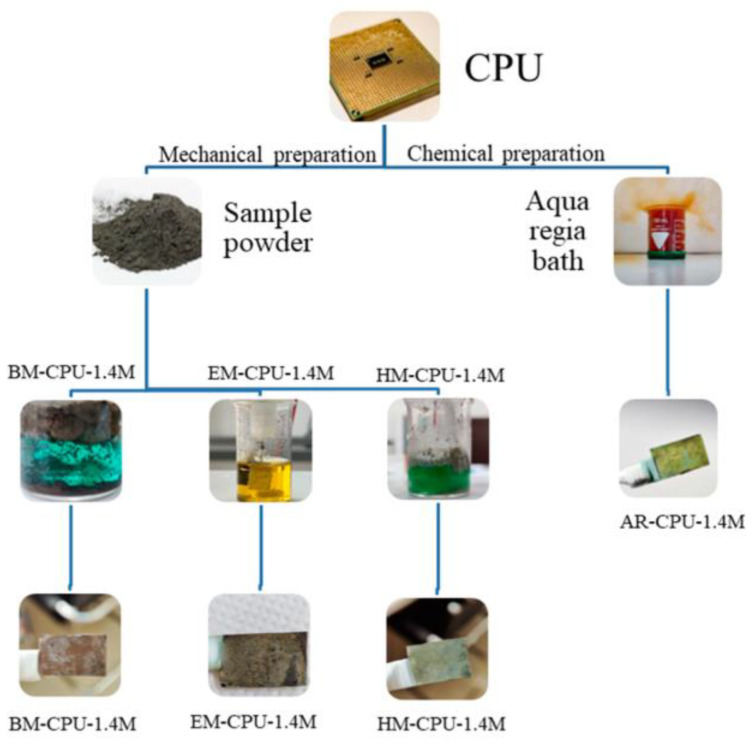
Schematic representation of the sample preparation procedure.

In this study, we began our research by comparing different methods for the preparation of samples from e-waste, which were to be used as electrodeposition baths to obtain catalyst deposits suitable for hydrogen production. Four different preparation techniques were applied, including one chemical method and three primarily mechanical preparation approaches described in previous sections and presented in Figure 1.

As previously described, after each mechanical treatment of the CPUs, the obtained grounded samples were sieved as described above and their weight was measured to determine the particle size and its proportion to the total volume [10]. Particle size mass fractions are presented in Table 1.

Based on the observed data, the highest proportion of fine particles was derived from the ball milling technique. It is crucial to note that, even after a duration of 6 h, complete grinding of the entire sample was not achieved. This consideration, in tandem with the subsequent electrochemical characterization, suggests caution in interpreting the results derived from this sample preparation method. Specifically, for the purpose of electrode preparation for hydrogen production in alkaline electrolysis, this method may not be the most optimal. The particle size, in conjunction with consistent stirring during the electrolytic process, plays a pivotal role in the preparation of our electrodeposition bath. Utilizing a magnetic stirrer for electrolyte preparation has been demonstrated to yield a narrow particle size distribution and maintain stable particle dispersions [11]. A combination of smaller particles and consistent stirring promotes stable particle dispersions in the bath, though it is less effective at disaggregating large particle clusters [12]. The distribution of mass percentages in the particle sizes of the ground samples in the other two milled samples (EM-CPU-1.4M and HM-CPU-1.4M) was relatively uniform, facilitating a more consistent comparison during electrochemical characterization.

### 3.2. Electrochemical Testing

**Figure 2:**
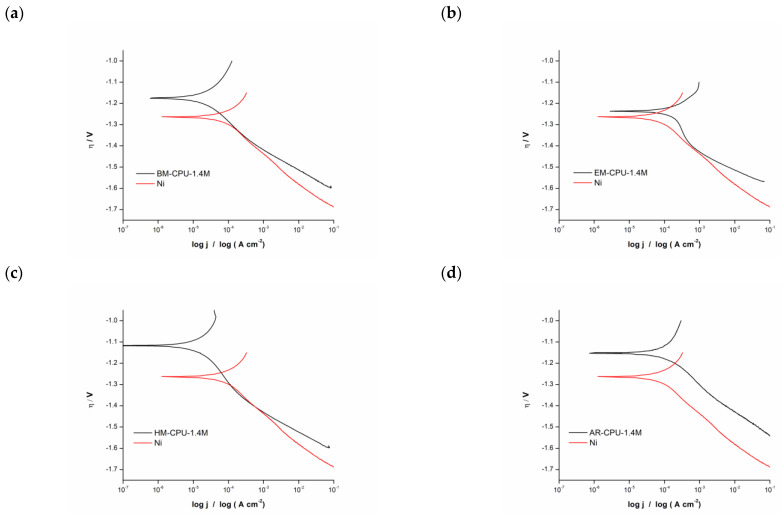
Polarization curves for HER in 6 M KOH with nickel electrode and electrodes coated with the samples: (**a**) BM-CPU-1.4M, (**b**) EM-CPU-1.4M, (**c**) HM-CPU-1.4M, (**d**) AR-CPU-1.4M.

The kinetics and mechanisms of the hydrogen evolution reaction (HER) on cathodes electrodeposited from solutions containing the subject samples were analyzed via Tafel analysis and Electrochemical Impedance Spectroscopy (EIS). Polarization curves and EIS spectra were acquired in a 6 M KOH standard electrolyte solution comparing bare nickel electrodes to those coated with the samples under study. These polarization curves are presented in Figure 2.

A subsequent Tafel analysis was performed on the captured polarization curves. For the analyses, two data sets were examined: one representing the bare metallic Ni electrode and another for the Ni electrode coated with the electrodeposited catalyst. A noteworthy observation across all samples was that the polarization curves for electrodes with the catalytic coating revealed a positive shift, gravitating towards less negative potential values in comparison with the pristine Ni electrode.

In the Tafel plot, a linear relationship emerges between the logarithm of the current and the overpotential. The curve’s slope, known as the Tafel slope, provides insight into the reaction’s activation energy, and, consequently, its rate. A pronounced slope suggests a higher requisite activation energy, implying a diminished reaction rate [13].

The derived Tafel slope values for each sample are detailed in Table 2. Leveraging these values, a rudimentary reaction mechanism is inferred. Broadly, three mechanisms can be categorized: activation-controlled, diffusion-controlled, and mixed-controlled reactions. Within this study’s context, slope values spanning between 90 and 120 mV/dec were observed, correlating with a mixed-controlled reaction mechanism. Such a mechanism amalgamates aspects of both activation- and diffusion-controlled processes [14].

The exchange current density, indicative of an electrode’s propensity to facilitate an electrochemical reaction, was also evaluated. A heightened exchange current density signifies enhanced electrode surface activity. The data indicate that the AR-CPU-1.4M electrode possesses the maximal exchange current density. This underscores that this particular coating manifests significantly superior catalytic activity compared with other electrode coatings and the uncoated Ni electrode.

**Figure 3:**
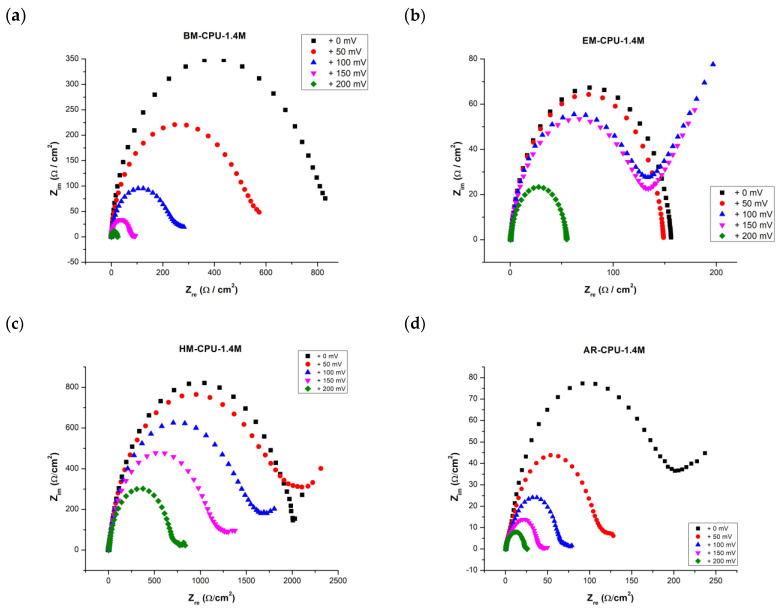
Impedance diagrams (Nyquist diagrams) of the coated electrodes noted on each graph recorded at overpotentials ranging between 0 mV to 200 mV: (**a**) BM-CPU_1.4M coating, (**b**) EM-CPU-1.4M coating, (**c**) HM-CPU-1.4M coating and (**d**) AR_CPU_1.4M coating.

Enhanced understanding of the hydrogen evolution electrochemical reaction mechanism was facilitated by capturing electrochemical impedance spectra for electrodes coated in a 6 M KOH standard electrolyte solution. The impedance plots, or Nyquist diagrams, for these coated electrodes are illustrated in Figure 3.

In the Electrochemical Impedance Spectroscopy (EIS) study, spectra were recorded at ambient temperatures over a range of overpotentials between 0 mV and 200 mV. The accompanying Nyquist plots, depicted in the aforementioned figure, manifest as a distorted semicircle. This pattern suggests that the Randles equivalent circuit may be apt for modeling and discerning other EIS-related attributes.

**Figure 4:**
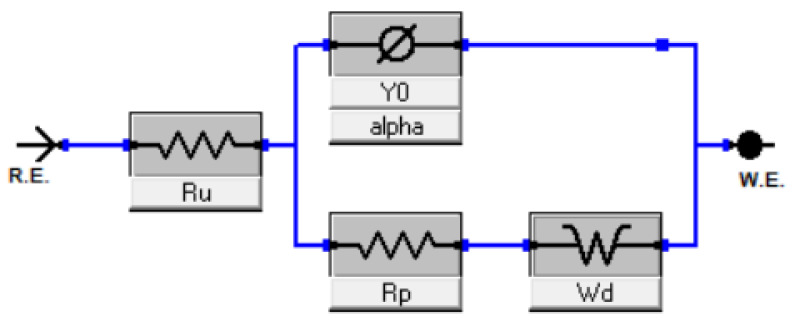
The electrical circuit used to fit the EIS spectra.

Notably, as the overpotential range shifts between 100 mV and 200 mV, a diffusion tail emerges, giving rise to what is termed the Warburg impedance. This impedance exhibits a frequency dependence; at lower frequencies, the Warburg impedance increases due to the extended diffusion distance of the reactants. The acquired EIS spectra were modeled using a hybrid Randles circuit, incorporating a constant phase element (CPE) in lieu of the double layer capacitance (Figure 4). This substitution accounts for the non-ideal behavior of the double layer. Additional parameters integrated in the model are the diffusion coefficient, electrolyte resistance, and charge transfer resistance. These parameters facilitated the derivation of the electrode surface roughness factor (σ) and the single time constant (τ). For samples BM-CPU-1.4M and AR-CPU-1.4M, the roughness factor was approximately 50, which correlates with the electrode’s active surface area. Consequently, the active surface area post electrodeposition was approximately 50 times greater than the electrode’s geometric surface. Concurrently, the double layer capacitance showcased favorable metrics, suggesting potential applications for this e-waste-derived material in capacitors and supercapacitors (refer to Table 3). While the roughness factor and double-layer capacitance values for the ball-milled and aqua regia-treated samples are relatively congruent, the AR-CPU-1.4M sample demonstrates superior attributes. A distinguishing feature between the two samples, underscoring the enhanced properties of the AR-CPU-1.4M sample, is its substantially lower charge transfer resistance across all examined overpotentials compared with its counterparts [15,16,17].

### 3.3. Elemental Composition and Morphological Characteristics

The elemental composition and morphological properties of both the electrolytic bath and the electrodeposited coating of the AR-CPU-1.4M electrode were assessed using scanning electron microscopy (SEM), Energy-Dispersive X-ray Spectroscopy (EDS), and Inductively Coupled Plasma Optical Emission Spectroscopy (ICP-OES). The AR-CPU-1.4M electrode was chosen for detailed examination due to its superior electrochemical performance in the hydrogen evolution reaction, as evidenced by the Tafel and EIS analyses.

After electrochemical characterization, the surface of the electrode was analyzed with scanning electron microscopy with Energy-Dispersive X-ray Spectroscopy (SEM/EDX).

**Figure 5:**
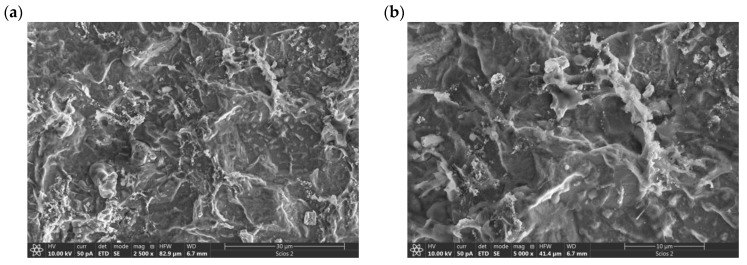

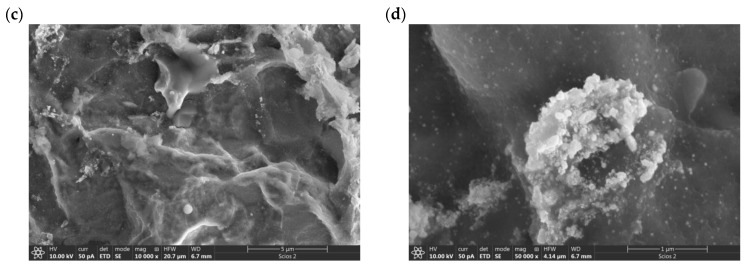
SEM micrographs of catalyst surface of electrodeposited AR-CPU-1.4M sample at: (**a**) 2500×, (**b**) 5000×, (**c**) 10,000×, (**d**) 50,000× magnification.

**Figure 6:**
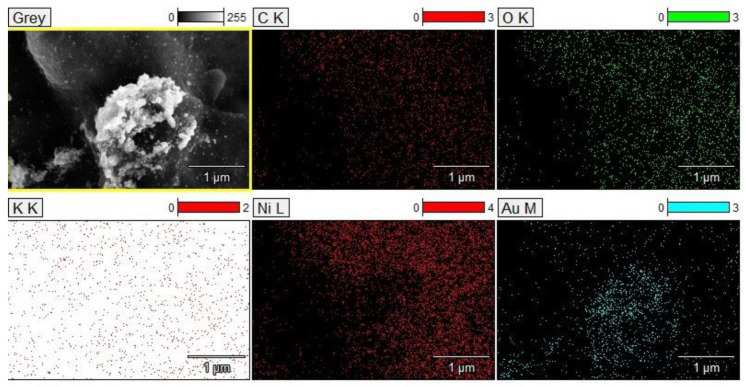
EDS mapping with element distributions of the electrodeposited coating.

SEM micrographs of the electrodeposited coating display a surface characterized by a distinctive rough texture and a commendable surface evolution. A significant feature that can be observed in the micrographs is the tendency of the deposited metals to aggregate. Particularly in Figure 5d, formations reminiscent of gold ‘islands’ are apparent. EDS analysis confirms that these ‘islands’ are formed by gold particles (Figure 6).

Notably, these elemental structures are nanoscale in nature; the gold particles exhibit diameters ranging from 10 to 20 nm. Under high magnification in the SEM micrographs, the presence of these electrodeposited gold particles is unequivocally discernible, which is further corroborated by EDS analysis.

Utilizing EDS analysis, the elemental composition of the electrodeposited coating was investigated. As delineated in Table 4, copper and gold prominently dominate the coating’s composition. Additionally, notable quantities of silver and aluminum were observed. These observations are consistent with our anticipations, reflecting the intrinsic composition of the CPUs initially used for dissolution.

**Figure 7:**
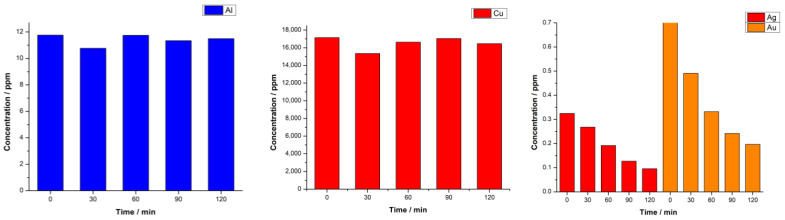
ICP-OES analysis of AR-CPU-1.4M deposition bath throughout deposition.

Through ICP analysis, we sought to monitor the fluctuations in metal concentrations throughout the electrodeposition process. The results, presented in Figure 7, distinctly show the successful detection of our target elements, with their concentrations registering a marked decrease as the process progressed. These data further elucidate the prominent copper deposition observed in the EDX analysis, attributable to the high concentration of dissolved copper present in the electrodeposition bath.

## 4. Conclusions

One of the foremost considerations for the optimal harnessing of metals from e-waste, particularly CPUs, rests upon the process of sample preparation. Our methodologies encompassed different grinding techniques aimed at achieving a refined particle size distribution and improved surface area. The inherent distinction between these techniques revealed crucial findings. Specifically, the ball mill approach facilitated a particle fraction that was most conducive for further processing in comparison with other protocols. Nonetheless, it is vital to note that, even after exhaustive grinding durations, the entirety of the CPU material remained incompletely processed. This sheds light on the inherent challenges tied to achieving optimal particle morphology and, by extension, reiterates the importance of refining our sample preparation approaches. Thus, the relationship between particle size distribution and electrochemical behavior underscores the need for meticulous attention during this phase of the process.

The hydrogen evolution reaction (HER) serves as an informative metric for gauging the efficiency of our electrodeposited coatings. Notably, the AR-CPU-1.4M electrode, amongst our samples, demonstrated superior electrocatalytic properties, which warranted further morphological and elemental exploration. SEM micrographs of this particular electrode depicted a surface rich in topographical details. These findings were reinforced by the EDS and ICP analyses, which showcased a pronounced influence of gold and copper on the coating’s composition. The results resonated with the inherent composition of CPUs, providing a rationale for the element clustering observed in our analyses. Particularly notable was the nanoparticulate nature of the gold deposits and their impact on the catalytic activity of the electrode.

Furthermore, a systematic examination of the deposition bath unveiled a high concentration of dissolved copper, which offered insights into the prominent copper deposition observed in the EDX analysis. Given the pivotal role of copper in hydrogen evolution and the established correlations between metal concentration and deposition outcomes, these observations afford valuable perspectives for tailoring our sample preparation techniques and electrodeposition protocols.

Our foray into the realm of harnessing e-waste as a repository for catalytic metals, especially for hydrogen production, has been insightful. The emphasis on nuanced sample preparation has reiterated its significance in governing the efficiency of the subsequent electrochemical processes. The AR-CPU-1.4M electrode, in particular, has underscored the potential that lies within optimally processed e-waste. The correlation between the detailed morphological and elemental characteristics and the electrocatalytic activity of the electrode demonstrates the interconnectedness of our preparation techniques and the resultant electrochemical behavior. It is, therefore, evident that the sustainable and efficient utilization of e-waste, especially in the context of hydrogen production, mandates an integrative approach that bridges meticulous sample preparation, in-depth morphological understanding, and electrochemical expertise. In advancing the narrative of the circular economy, this work has not only highlighted the vast potential of e-waste but has also illuminated pathways to optimally harness it.

## Figures and Tables

**Table 1:** Particle size mass percent in the mechanically prepared samples.

Particle Size	BM-CPU-1.4M (%)	EM-CPU-1.4M (%)	HM-CPU-1.4M (%)
>1 mm	7.77	13.63	12.63
>500 μm	1.35	27.58	25.25
>250 μm	3.23	23.39	25.92
>125 μm	28.44	17.71	15.55
<125 μm	59.22	17.69	20.65

**Table 2:** Kinetic parameters for Ni electrodes in 6M KOH and for coated electrodes.

Sample	Tafel Slope (mV/dec)	Exchange Current Density (A cm^−2^)
BM-CPU-1.4M	94.4	5.637 × 10^−6^
EM-CPU-1.4M	104.7	28.420 × 10^−6^
HM-CPU-1.4M	100.0	1.795 × 10^−6^
AR-CPU-1.4M	110.0	29.790 × 10^−6^
Ni	120.0	23.450 × 10^−6^

**Table 3:** EIS parameters for all electrodeposited samples.

T (K)	−η (mV)	R_e_ (Ω cm^2^)	R_ct_ (Ω cm^2^)	α	C_dl_ (μF cm^−1^)	σ	τ (s)
BM-CPU-1.4M							
298.15	0	0.518	790.0	0.937	1121.4	56	0.886
	50	0.513	510.1	0.928	1247.9	62	0.637
	100	0.511	228.5	0.925	1231.6	62	0.282
	150	0.581	76.5	0.932	802.1	40	0.061
	200	0.514	22.5	0.955	449.9	22	0.010
EM-CPU-1.4M							
298.15	0	0.561	150.2	0.912	406.2	20	0.061
	50	0.559	140.4	0.916	401.9	20	0.056
	100	0.557	121.2	0.919	392.9	20	0.048
	150	0.542	123.3	0.891	356.6	18	0.044
	200	0.546	52.3	0.909	227.7	11	0.012
HM-CPU-1.4M							
298.15	0	0.575	1199.1	0.869	139.2	7	0.167
	50	0.568	1858.0	0.870	129.6	6	0.241
	100	0.565	1593.0	0.873	117.5	6	0.187
	150	0.565	1161.0	0.879	103.9	5	0.121
	200	0.561	721.3	0.887	86.3	4	0.062
AR-CPU-1.4M							
298.15	0	0.545	206.3	0.779	1269.4	63	0.262
	50	0.547	117.8	0.776	1037.2	52	0.122
	100	0.544	67.5	0.773	788.6	39	0.053
	150	0.545	39.8	0.785	565.1	28	0.022
	200	0.557	22.7	0.819	373.3	19	0.008

**Table 4:** EDS elemental analysis of electrode surface for sample AR-CPU-1.4M.

Element	Weight %	Atom %
Al	0.554	1.442
Cu	83.921	92.839
Ag	0.607	0.394
Au	14.918	5.324

## Data Availability

Not applicable.
